# How predictable are mass extinction events?

**DOI:** 10.1098/rsos.221507

**Published:** 2023-03-15

**Authors:** William J. Foster, Bethany J. Allen, Niklas H. Kitzmann, Jannes Münchmeyer, Tabea Rettelbach, James D. Witts, Rowan J. Whittle, Ekaterina Larina, Matthew E. Clapham, Alexander M. Dunhill

**Affiliations:** ^1^ Institute for Geology, University of Hamburg, Hamburg, Germany; ^2^ School of Earth and Environment, University of Leeds, Leeds, UK; ^3^ Department of Biosystems Science and Engineering, ETH Zürich, Basel, Switzerland; ^4^ Computational Evolution Group, Swiss Institute of Bioinformatics, Lausanne, Switzerland; ^5^ Potsdam Institute for Climate Impact Research (PIK)—Member of the Leibniz Association, Potsdam, Germany; ^6^ Institute of Physics and Astronomy, University of Potsdam, Potsdam, Germany; ^7^ Institute of Geosciences, University of Potsdam, Potsdam, Germany; ^8^ GFZ German Research Centre for Geoscience, Potsdam, Germany; ^9^ Department of Computer Science, Humboldt-Universität zu Berlin, Berlin, Germany; ^10^ Permafrost Research Section, Alfred Wegener Institute Helmholtz Centre for Polar and Marine Research, Potsdam, Germany; ^11^ Bristol Palaeobiology Research Group, School of Earth Sciences, University of Bristol, Bristol, UK; ^12^ British Antarctic Survey, High Cross, Cambridge, UK; ^13^ Department of Earth Sciences, University of Southern California, Los Angeles, CA, USA; ^14^ Jackson School of Geosciences, University of Texas, Austin, Texas, USA; ^15^ Department of Earth and Planetary Sciences, University of California, Santa Cruz, CA, USA

**Keywords:** mass extinction, machine learning, fossil, end-Permian, end-Triassic, end-Cretaceous

## Abstract

Many modern extinction drivers are shared with past mass extinction events, such as rapid climate warming, habitat loss, pollution and invasive species. This commonality presents a key question: can the extinction risk of species during past mass extinction events inform our predictions for a modern biodiversity crisis? To investigate if it is possible to establish which species were more likely to go extinct during mass extinctions, we applied a functional trait-based model of extinction risk using a machine learning algorithm to datasets of marine fossils for the end-Permian, end-Triassic and end-Cretaceous mass extinctions. Extinction selectivity was inferred across each individual mass extinction event, before testing whether the selectivity patterns obtained could be used to ‘predict’ the extinction selectivity exhibited during the other mass extinctions. Our analyses show that, despite some similarities in extinction selectivity patterns between ancient crises, the selectivity of mass extinction events is inconsistent, which leads to a poor predictive performance. This lack of predictability is attributed to evolution in marine ecosystems, particularly during the Mesozoic Marine Revolution, associated with shifts in community structure alongside coincident Earth system changes. Our results suggest that past extinctions are unlikely to be informative for predicting extinction risk during a projected mass extinction.

## 1. Introduction

Highly elevated extinction rates in many clades, alongside multiple other lines of evidence, indicate that we are currently witnessing a biodiversity crisis (e.g. [1–7]). The major drivers of extinction today, including climate change, habitat loss, pollution, invasive species and over-exploitation, are human-induced [8–14]. These drivers of extinction, particularly climate change and habitat loss, are also associated with mass extinction events and rapid climate warming events (i.e. ‘hyperthermals’; see [15]) in geological history, albeit previously caused by Earth system phenomena such as volcanic activity or bolide impacts [7,16]. There are also key differences in how these threats to biodiversity manifested themselves in the geological past compared with today, for example anthropogenic pollution is much wider in scope and includes synthetic substances, while the introduction of invasive species via human activities probably occurs at much broader spatial scales and at faster rates.

Traditionally, it is thought that life on Earth has experienced five mass extinction events [17], but the number of past mass extinctions has been called into question in more recent analyses (e.g. [18,19]), partly due to relative ambiguity in the definition of a mass extinction. A mass extinction event has previously been defined as a statistically distinct increase in the amount of extinction suffered by more than one geographically widespread higher taxon during a relatively short interval of geologic time, resulting in an at least temporary decline in standing diversity [17,20]. Regardless of this debate, of the traditional five mass extinctions, the three most recent (the end-Permian, end-Triassic and end-Cretaceous events, which occurred 252, 200 and 66 Ma, respectively) were the three most catastrophic, both taxonomically and ecologically [19,21]. Given the kill mechanisms of these events that overlap to varying degrees (despite different triggers of cascading environmental changes), do these events have shared extinction selectivity patterns and can these mass extinction events in deep time, therefore, be used as analogues for the modern biodiversity crisis, informing our predictions concerning selectivity during a projected mass extinction over the next century?

The end-Permian and end-Triassic mass extinctions were coincident with the eruption of the Siberian Traps and Central Atlantic Magmatic Province Large Igneous Provinces (LIPs), respectively. These LIPs are associated with cascading environmental changes hypothesized to have driven the extinctions. Climate warming resulting from greenhouse gas emissions subjected animals to thermal stress, and in the oceans, this was compounded by the additional deleterious effects of deoxygenation and ocean acidification [16,22]. In addition, it is thought that toxic metal emissions from the LIPs accumulated to lethal levels that also contributed to heightened mortality rates during these mass extinctions [16]. The cause and drivers of the end-Cretaceous mass extinction have been more heavily debated, with the eruption of the Deccan Traps LIP and the bolide that created the Chicxulub impact crater both occurring at a similar time to the mass extinction event [23–26]. The bolide impact is, however, generally favoured as the cause by the research community (e.g. [25–28]). A large extraterrestrial impact results in a different suite of environmental changes to an LIP: thermal stress via both a pulse of extreme regional warming around the impact site and short-term (decadal) extreme global climate cooling, followed by ocean acidification and a primary productivity decline over longer time scales [27].

It is becoming increasingly accepted that the ‘winners’ and ‘losers’ during mass extinction events are not determined at random; instead, the possession of certain functional characteristics or traits may influence the probability of a clade becoming extinct during a mass extinction, an effect described as extinction selectivity (e.g. [29–34]). The importance of many characteristics in selectivity during mass extinction events has previously been debated, including biological traits such as body size, diet and motility [29,32–35], and geographical ‘traits’ such as range size and occupied latitude [29,33,36–41]. Comparing extinction selectivity patterns between mass extinction events may, therefore, provide insight into the consistency of selectivity signals during times of heightened extinction rates. Only a few comparative extinction selectivity studies have compared the end-Permian, end-Triassic and end-Cretaceous mass extinction events [38,42–44]. These studies investigated extinction selectivity using a single functional trait, or a binary classification of functional traits. It has, however, been shown that more detailed analyses of extinction selectivity are possible using a multivariate framework. Both statistical methods and machine learning algorithms offer the potential to investigate extinction selectivity across more dimensions, such as using non-binary classifiers of functional traits (e.g. [33,45,46]), and quantitatively testing extinction selectivity patterns across a suite of traits between different time intervals (e.g. [46–48]).

To compare extinction selectivity between mass extinctions and the other time intervals (here referred to as ‘background’ intervals), we assessed the traits possessed by genera which became extinct during each event. This was conducted by building a functional trait-based model of extinction risk for each mass extinction, using a machine learning algorithm (gradient boosting on decision trees). The fit between each of these models and the other two extinction events was then tested quantitatively, to determine how transferable these models are, and therefore the extent to which selectivity regimes were comparable between events. A functional trait-based approach was used because the functional composition and the presence/absence of functional groups in the ocean have evolved over time. By uncovering the commonalities of extinction selectivity, the machine learning algorithms can use extinction selectivity from functional groups that became extinct to inform predictions, and can make predictions about functional groups that did not exist at previous extinction events. This approach also enabled the evaluation of whether past extinction events could be used to make predictions about a projected future mass extinction event, even though the functional composition of modern oceans are different to those of the past.

## 2. Material and methods

### 2.1. Data

To obtain datasets to investigate extinction selectivity for the end-Permian, end-Triassic and end-Cretaceous mass extinction events, we accessed species-level occurrences of marine invertebrates and conodonts from the Paleobiology Database (https://paleobiodb.org), downloaded in August 2022. Occurrences indicate the presence of a taxon in a given locality (collection) during a specified interval of geological time. To identify which taxa went extinct or survived during these different events, we downloaded datasets from two time intervals: (i) Capitanian to Toarcian (264.3–174.1 Ma) and (ii) Cenomanian to Eocene (100.5–33.9 Ma). The wide breadths of these time intervals were chosen to avoid generating edge effects within our extinction events of interest (*sensu* [49]). The clades represented within the datasets were the Annelida, Arthropoda, Brachiopoda, Bryozoa, Cnidaria, Echinodermata, Mollusca, Porifera and Conodonta.

The occurrences were manually vetted to ensure that individual species were not represented within multiple genera due to taxonomic synonymy, in which case the most up-to-date generic identification of the species was followed. For example, in the raw dataset, the species *Oliva mitreola* was also included in the genera *Olivancillaria*, *Olivella* and *Pseudolivella*, but these were all manually amended to *Oliva*. In addition, subgenera were checked to ensure that they were not included as both genera and subgenera. Typographic errors were also vetted at both the genus and species level to ensure that taxa did not appear with multiple spellings. To calculate the stratigraphic range of each genus, occurrences of genera with open nomenclature (“”, ?, aff., cf., informal) were excluded, as well as occurrences that were not identified to species level. The latter was done to avoid taxonomic artefacts in the reassignment of species: when a species is reassigned to a new genus, its occurrences are moved to that genus, whereas a genus-level assignment is left in the original genus. Freshwater genera were also removed. Following this, the range of each genus was determined based on their first and last observed occurrences within these datasets.

To investigate extinction selectivity for each event, the genera known from the Changhsingian (254–252.17 Ma), Rhaetian (208.5–201.3 Ma) and Maastrichtian (72.1–66 Ma) stages were selected to investigate the end-Permian, end-Triassic and end-Cretaceous events, respectively. For each event, each genus was classified as going extinct (1) or surviving (0) based on whether or not the range of the genus crossed the stage boundary. Genera with less than three occurrences were omitted from the analysis, as the timing of their disappearance from the database record is more likely to be an artefact of poor taxon sampling than an indicator of extinction. Lazarus genera (those that disappear from the fossil record for one or more periods, only to reappear later) were also excluded because their post-extinction ‘traits’ (e.g. geographical range) are obscured by the lack of data, which would have a negative impact on the robustness of the trained classifiers’ decisions. After data cleaning, extinction selectivity was determined based on 635, 649 and 1358 marine genera for the end-Permian, end-Triassic and end-Cretaceous events, respectively. To investigate if the mass extinction events shared similar extinction selectivity patterns to ‘background’ time intervals, we also created classifications for each of the geological stages included in our datasets.

### 2.2. Ecospace assignments

We characterized each genus according to seven ecological traits: tiering (life position relative to the sediment–water interface) [50], motility [50], feeding [50], respiratory protein [46], reproduction [51], mineralogy [46] and skeletal physiology [31,44] (table 1). These assignments were based on the primary literature and traits of extant relatives, referring to the ecological attribute during a taxon’s adult life stage [33,39,52–54]. Taxa that occupied multiple functional groups were weighted equally between the two groups: for example, a genus described as a grazer was weighted as 1 for that group, whereas a genus classified as both a predator and grazer was weighted as 0.5 for each of the two functional traits. Number of constituent species and geographical range size have also been suggested to represent ‘traits’ of genera and potentially good predictors of extinction risk (e.g. [55]). To estimate geographical range size, we followed Payne & Finnegan [38] and tabulated the number of tectonic plates (‘geoplates’ in the raw download) on which each genus occurred in each time bin. To estimate the number of species within a genus, the number of named species for each genus in each time bin was tabulated. Occurrences without a species name (e.g. sp. or spp.) were assumed to represent a single species in addition to those that were named. These traits were chosen because they have been shown to reveal particular selectivity signals when extinctions are a consequence of climate change (e.g. [33,43,46,51,56]), and can be applied to all the groups investigated in this study.

**Table 1.** Ecological categories used in this study to explore extinction selectivity. See methods for further details.

Ecological category	Ecological category
*Tiering*	*Mineralogy*
1. pelagic	1. aragonite
2. erect	2. low-Mg calcite
3. epifaunal	3. high-Mg calcite
4. semi-infaunal	4. bimineralic
5. shallow infaunal	5. phosphatic
6. deep infaunal	6. chitin
*Motility*	7. gorgonin
1. fast, motile	8. silica
2. slow, motile	9. soft-bodied
3. facultative, unattached	*Skeletal physiology*
4. facultative, attached	1. Group I
5. stationary, unattached	2. Group II
6. stationary, attached	3. Group III
*Feeding*	*Reproduction*
1. suspension feeder	1. non-broadcaster
2. surface deposit feeder	2. intermediate
3. miner	3. broadcaster
4. grazer	*Respiratory protein*
5. predator	1. hemerythrin
6. photo-/chemosymbiosis	2. haemocyanin
*Geographical range* (continuous)	3. haemoglobin
*Number of species* (continuous)	4. other

Even though the number of total and pre-extinction occurrences were calculated for each genus and event, and have been included in previous extinction selectivity studies as indicators of sample comparability (e.g. [32]), they were not included in the analyses here for two reasons. Firstly, the total number of occurrences is heavily influenced by sampling bias and is, therefore, not a robust indicator of abundance. Secondly, using such a ‘trait’ would also harm the robustness of the trained classifiers’ decisions on the remaining traits and compromise their inferred role in determining extinction selectivity. This is due to the earlier removal of genera with less than three total occurrences from the dataset: if a genus has less than three pre-extinction occurrences, it must have at least one or two additional occurrences after the event, and, therefore, be a survivor.

### 2.3. Gradient-boosted trees

We used an open source library for gradient boosting on decision trees (CatBoost [57]) as the model for predicting survivorship, implemented in Python. Gradient-boosted trees are a form of machine learning that works by progressively training more complex models to maximize the accuracy of predictions [57]. Gradient-boosted trees have proved to be well suited for determining extinction selectivity in previous studies [46,47,55]. The CatBoost algorithm provides a measure of each feature’s importance in the model, here indicating which traits had the greatest overall effect on the classification decision. This importance measure is implemented as the average change in the model’s prediction value upon a change in the trait value, referred to as *PredictionValueChange*. The feature importances are non-negative, and normalized to sum to 100. For each time interval, we trained 10 models on randomly chosen train/test splits of the genera with the ratio 8 : 2. All results reported are from the test sets, unless indicated otherwise. For our cross-extinction analyses, we trained the model on 80% of genera from one extinction and tested it on all genera from another extinction.

All three mass extinction events were relatively balanced between extinct and non-extinct genera, with extinction proportions between 0.53 (end-Cretaceous event) and 0.74 (end-Permian event). We, therefore, do not expect there to have been a negative impact on training adequacy from a class imbalance for the individual extinction events. However, the difference in extinction rate between extinction events needed to be taken into account for the cross-extinction analyses. To this end, we evaluated the performance of our models using receiver operating characteristic (ROC) curves, and their area under the curve (AUC). The ROC/AUC only depend on the order of the samples, given by their extinction likelihood predicted from the CatBoost, and do not take into account their absolute likelihood values. As a result, they are generally insensitive to the different prior/marginal extinction likelihoods. Moderate differences in extinction rate between our datasets should thus not distort the ROC/AUC.

To find the best configuration of the CatBoost model, we conducted a hyperparameter optimization, using a wide grid search on the most important parameters of the model (tree depth, learning rate and L2 regularization). The optimization of hyperparameters did not, however, improve test performance significantly above the performance using default parameters (see electronic supplementary material, figure S2). The generally low impact of the hyperparameters on test performance may be attributed to several reasons. First, the CatBoost algorithm is stable with respect to hyperparameter selection, i.e. algorithm performance should only be mildly affected by different hyperparameters [57]. Second, the complexity of the prediction task results is, to a large extent, due to the fact that not all genera in a functional group have the same status of survival/extinction, but the model can only assign one prediction to the functional group. This limits the overall performance. Third, the total dataset size is relatively small compared with those typically used for similar machine learning analyses. The amount of information that can be learned from the data is, therefore, restricted, and adjusting the model hyperparameters cannot yield better performance. As a result, we opted to use the default hyperparameters from CatBoost for our experiments. To quantify the extent of overfitting, we compared the average difference in AUC score between the training and test sets for each extinction event. This was 4.5 percentage points (pp) for the end-Permian, 10.0 pp for the end-Triassic and 6.6 pp for the end-Cretaceous. These values indicate only a modest level of overfitting.

To investigate if the mass extinction events had unique extinction selectivity signals, or were simply an intensification of long-term Phanerozoic extinction patterns, we implemented a principal component analysis (PCA) on the trait importances for each geological stage included in the datasets. This also meant that we could compare the mass extinction selectivity signals with those of several other well-known extinction and hyperthermals, such as the Palaeocene–Eocene thermal maximum (PETM).

### 2.4. Shapley additive explanations

To make the predictions from the CatBoost models easier to interpret, we used Shapley additive explanations (SHAP [58]). SHAP values provide a unified framework for interpreting the feature importance of model predictions. The concept is based on the Shapley values from cooperative game theory, where the distribution of a payout is calculated based on the individuals’ contributions to the size of the payout [59]. In the context of model predictions, this translates to quantifying the contribution of each feature towards the model output for each individual sample [58]. Positive SHAP values indicate a positive contribution towards the prediction, in this case extinction, while negative values point towards survival. Calculating SHAP values provides us with both global and local interpretations. Global insights let us quantify the (positive or negative) impact of any trait and its constituent categories over the entire dataset for each mass extinction, facilitated with SHAP summary plots. For local insights, we can also compute SHAP values for individual traits, visualized with force plots. For this, we calculate the base value, which corresponds to the average of all predictions from the dataset. We then calculate the prediction associated with a functional group by adding all calculated feature contributions to this base value. Our SHAP values were trained on a CatBoost model for the entire dataset; this is in contrast to the data splits used in the rest of the analyses, which enable a comparison of model performance. The SHAP values, therefore, reflect a higher proportion of the available data. Comparing SHAP values for ecological traits enables us to identify patterns of selectivity within the dataset.

## 3. Results

### 3.1. Principal component analysis

**Figure 1.**
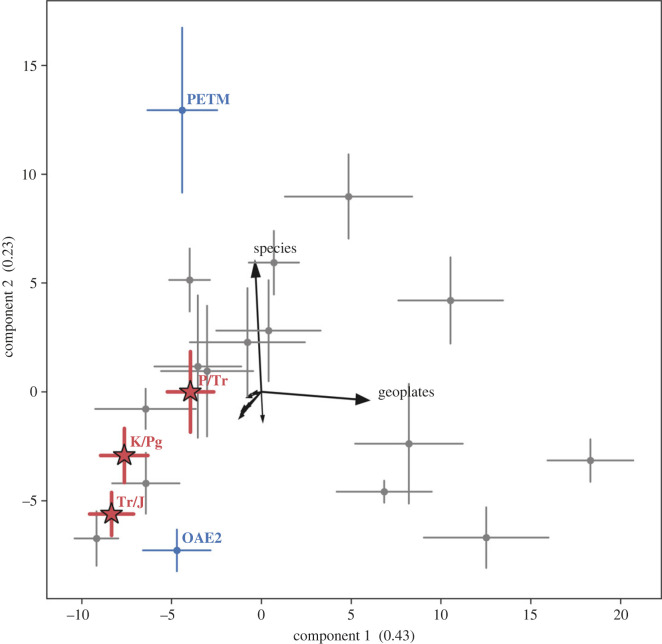
Principal component analysis of the feature importances generated by the CatBoost algorithm for each investigated geological stage. The points for each stage show the mean trait importance over 10 splits and the bars show ±1 s.d. The explained variance for each axis is provided in brackets. The three mass extinction events are highlighted in red with stars: P/Tr = end-Permian event, Tr/J = end-Triassic event, K/Pg = end-Cretaceous event. We further highlight the end-Cenomanian event (OAE2) and the Palaeocene–Eocene thermal maximum (PETM). The black arrows indicate the composition of the PCA components, with each arrow indicating one feature. The longest PCA arrows have the greatest correlation with the PCA components. Only the features referenced in the text are labelled.

The PCA of feature importances recognized by the CatBoost algorithm shows that PCA axes 1 and 2 are strongly influenced by the number of occupied geoplates (a proxy for geographical range size) and within-genus species richness, respectively, and are alone the best predictors of extinction for most of the investigated geologic stages (figure 1 and table 2). The exceptions are the extinction selectivity for the end-Permian, end-Triassic, end-Cenomanian (OAE2) and end-Cretaceous events, which cluster in the double negative quadrant of the PCA; during these events, geographical range size and species richness do not appear to have had a strong influence on extinction selectivity. In addition, the stages capturing the aftermath of the end-Permian (Induan and Olenekian) and end-Triassic (Hettangian and Sinemurian) mass extinctions also do not correspond to this otherwise dominant selectivity signal. This suggests that extinction selectivity during these mass extinction events (and their aftermaths) was not simply an intensification of long-term Phanerozoic extinction patterns.

**Table 2.** The explained variance of each feature per component in the PCA figure 1. Values in italics show significance at *p* = 0.05.

	component 1	component 2
tiering	−0.17	−0.24
motility	−0.16	−0.10
feeding	−0.12	−0.11
respiratory protein	−0.02	−0.21
reproduction	−0.06	−0.01
mineralogy	−0.13	−0.03
skeletal physiology	−0.18	−0.19
number of geoplates	*0.93*	−0.04
number of species	−0.09	*0.92*

### 3.2. Extinction selectivity

**Figure 2.**
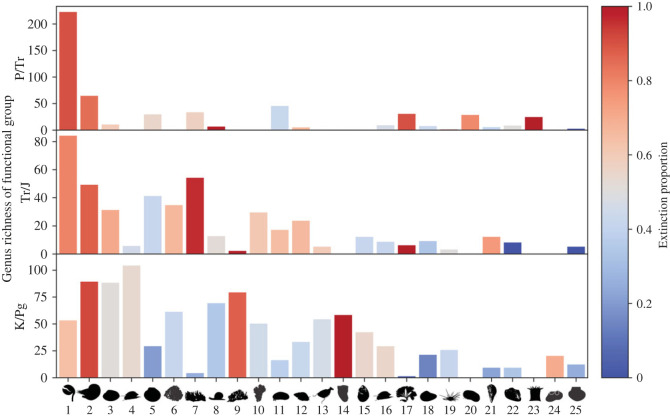
Number of genera within functional groups present in pre-extinction faunas, colour-coded with the proportion of genera that became extinct during the subsequent mass extinction. Vertically aligned bars denote the same functional groups in each extinction event, sorted by the total number of members in the functional groups across all three extinctions. The figure includes the 25 most prevalent functional groups of the 117 identified in the pre-extinction faunas. For a full overview of all functional groups, see electronic supplementary material, figure S1. P/Tr = end-Permian event, Tr/J = end-Triassic event and K/Pg = end-Cretaceous event.

The end-Permian, end-Triassic and end-Cretaceous events record unique extinction selectivity patterns compared with the other investigated stages of the Phanerozoic (figure 1). One hundred and seventeen unique functional groups (FG; combinations of traits) were recognized in the stages immediately prior to each of the three mass extinction events and are listed in electronic supplementary material, table S1. The number of genera distributed among these functional groups is different for each pre-extinction fauna, and 55 functional groups were unique to a single event (figure 2 and electronic supplementary material, figure S1). For example, FG14 is occupied by erect rudist bivalves that evolved after the Triassic, whereas FG23, which is represented by tabulate and rugose corals, goes extinct during the end-Permian mass extinction. It is also noteworthy that functional evenness increases with each subsequent event: the end-Permian pre-extinction fauna has a low functional evenness and is dominated by a single functional group (FG1; figure 2), while the end-Triassic pre-extinction fauna has a higher functional evenness, with dominance spread across more groups (in particular, FG1–FG2 and FG5–FG7). Finally, the end-Cretaceous pre-extinction fauna is dominated by even more functional groups (in particular, FG2–FG4 and FG8–FG9) and has a more even spread of genera among functional groups.

Functional groups present prior to all three events are particularly useful, as they clearly demonstrate the differences in extinction selectivity between events. Only 31 functional groups were present at all three mass extinction events. Six of these 31 functional groups had the same selectivity direction (more likely to survive versus more likely to become extinct) for all three extinction events. Only FG1 and FG2 were consistently at high risk of extinction at each event (figure 2). Interestingly, FG1 became more resistant to extinction, in that a smaller proportion of genera in the functional group went extinct at each subsequent extinction event (figure 2). The functional composition of marine ecosystems has, therefore, evolved and become more even between these events, which increases the complexity of comparing extinction selectivity.

#### 3.2.1. Machine learning

**Figure 3.**
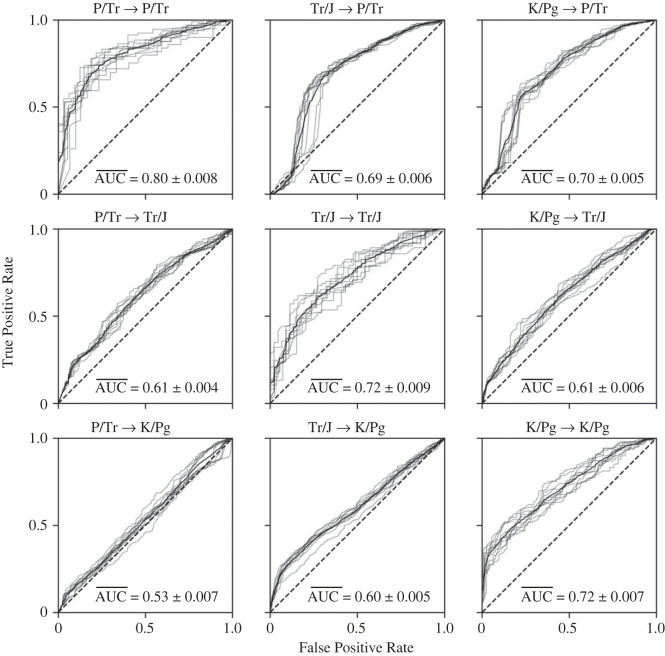
Receiver operating characteristic (ROC) curves showing CatBoost model performance trained and evaluated on each combination of extinction event datasets, with 10 models trained for each dataset combination. The labels should be understood as 〈training event〉 → 〈testing event〉. Each grey line represents one model; the continuous black line represents the average across the 10 models. The average area under the curve (AUC), rounded to two digits, is given at the bottom of each plot together with the standard error of the AUC. P/Tr = end-Permian event, Tr/J = end-Triassic event and K/Pg = end-Cretaceous event.

The area under the curve–receiver operating characteristic (AUC/ROC) (figure 3) visualizes the CatBoost model performance. This curve plots the true positive rate (proportion of genera correctly identified as having become extinct) against the false positive rate (proportion of surviving genera incorrectly classified as having become extinct) across different decision thresholds. An AUC of 1 indicates perfect classification, while an AUC of 0.5 indicates a random classification that has no utility. An AUC of greater than 0.7 is typically considered representative of a good model [60]. The resulting AUC/ROC curves show that the CatBoost algorithm is a good classification model for interpreting extinction selectivity for each event, with an AUC of 0.80, 0.72 and 0.72, for the end-Permian, end-Triassic and end-Cretaceous events, respectively (figure 3). The AUC/ROC also show that training the algorithm on one mass extinction event generally does not yield accurate predictions of extinction selectivity for other mass extinctions, with four of the six combinations having AUCs ranging from 0.53 to 0.61 (figure 3). This shows that extinction selectivity significantly varies between each mass extinction event, and that extinction selectivity at one mass extinction cannot be assumed to predict the selectivity of a different event. However, the algorithms trained on the end-Triassic and end-Cretaceous events, when applied to the end-Permian event, perform better than any other combination, with AUCs of 0.69 and 0.70, respectively (figure 3). This is because the end-Permian event was dominated by only a few functional traits that must be predicted to achieve an accurate result, and those groups typically suffered elevated extinction proportions during all three events.

**Figure 4.**
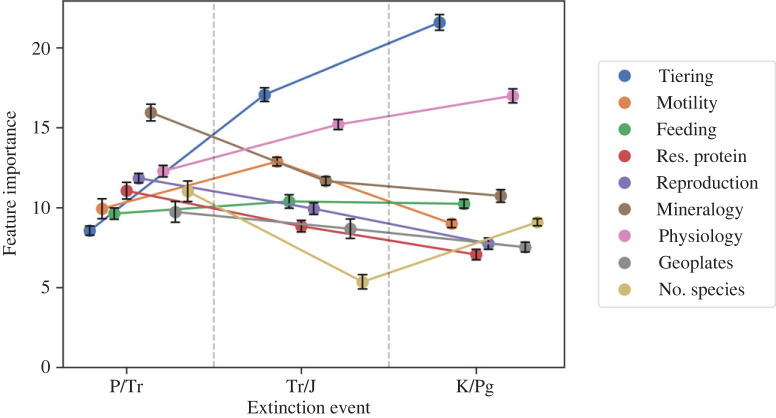
Relative importance of traits across the different mass extinction events as provided by the CatBoost algorithm. Results were obtained from averaging across the 10 models. Error bars show ±1 standard error from the mean estimate. P/Tr = end-Permian event, Tr/J = end-Triassic event and K/Pg = end-Cretaceous event.

Exploring the importance of different traits between mass extinction events further emphasizes that extinction selectivity varied between each event (figure 4). Comparing the trait importance values between each event, significant differences in their ranking and absolute values can be observed. For the end-Permian mass extinction, mineralogy was the single most important trait for predicting extinction selectivity (figure 4). At the end-Triassic mass extinction, tiering (which was one of the least important traits at the end-Permian event) became the most important feature, along with skeletal physiology. At the end-Cretaceous event, tiering was again the most important trait, and its importance continued to increase following the end-Triassic (figure 4). Skeletal physiology also remained important at the end-Cretaceous event (figure 4).

#### 3.2.2. Shapley additive explanations summary plots

SHAP values reveal the importance of dataset variables (traits) and categories (trait states) for predicting an outcome. Here, positive SHAP values suggest that the algorithm is using the trait state to predict that a genus will go extinct, whereas negative SHAP values indicate that the trait state will be used to predict that the genus will survive (e.g. figure 6). SHAP values near 0 will, therefore, have a smaller effect on the predictions than values near 1 or −1.

**Figure 5.**
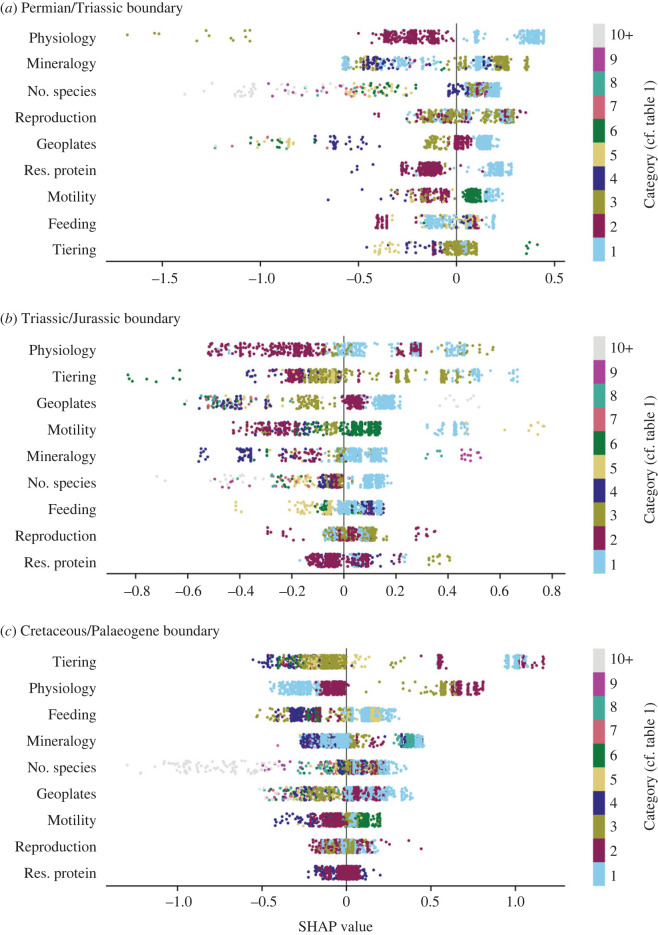
SHAP summary plot showing how the different categories of each ecological trait affect model predictions for the three extinction events. The horizontal location of the values shows whether a data point (genus) from the whole dataset is associated with a higher or lower prediction. The vertical ordering corresponds to the relative importance of each trait. The SHAP summary plot models were trained on all samples for each extinction (rather than the splits necessary for CatBoost). The points are coloured according to the categories given in table 1 for each trait. P/Tr = end-Permian event, Tr/J = end-Triassic event and K/Pg = end-Cretaceous event.

The SHAP summary plots (figure 5) show that selectivity patterns associated with a genus’s mineralogy and skeletal physiology, which are important predictors of extinction, are completely different for each event. For the end-Permian mass extinction, mineralogy is an important predictor of extinction; the SHAP values show that this is because genera that constructed their shell from low-Mg calcite mostly became extinct, and genera that constructed phosphatic shells, or did not have shells, were more likely to survive (figure 5 and table 1). This shows that it was not only FG1 that was selected against at the end-Permian event, as qualitatively observed in figure 2, but instead most functional groups with low-Mg calcite shells (figure 5). This finding demonstrates how a machine learning approach can reveal the commonalities of extinction selectivity between the different functional groups.

Skeletal physiology is an important predictor of extinction for both the end-Triassic and end-Cretaceous events. The SHAP values also show that even though skeletal physiology is not as important at the end-Permian as the other two events, there is still a clear pattern of extinction: for genera categorized as Group III (skeletons made of materials other than calcium carbonate), or genera categorized as Group I (skeletons made from calcium carbonate that was massive with respect to supporting organic tissue and formed from fluids minimally buffered by physiology), physiology is an important predictor of extinction, whereas genera categorized as Group II (calcium carbonate skeleton of moderate mass with respect to supporting living organic tissue and formed from fluids that are relatively well buffered) have SHAP values near 0 (figure 5), indicating that physiology is not a predictor of extinction for this trait category. The end-Cretaceous shows an inverse physiological selectivity pattern to the end-Permian: genera categorized as Group III were susceptible to extinction, and those categorized as Group I generally survived. The end-Triassic also shows a unique selectivity pattern, with genera categorized as Group III and categorized as Group I preferentially going extinct (figure 5). Overall, physiology is a good predictor of extinction, but patterns of selectivity for different categories within a trait were not always consistent between extinction events, which is why the machine learning algorithm cannot use one mass extinction to reliably make predictions about another.

The SHAP summary plots show that the most consistent selectivity patterns between all three events were for tiering and feeding. Tiering was one of the most important traits for selectivity during the end-Triassic and end-Cretaceous events (figure 4). The SHAP values show a clear pattern, with pelagic and erect taxa preferentially going extinct, while semi-infaunal, shallow infaunal and deep infaunal taxa preferentially survived. Another selective pattern seen in the SHAP values for all three events, but clearest at the end-Cretaceous, is that suspension feeders, predators and genera with symbiotic feeding styles were selected against (figure 5). Despite this clear selectivity pattern, feeding in general is not a good predictor of extinction selectivity.

#### 3.2.3. Shapley additive explanations force plots

One issue with comparing extinction selectivity between events is the different functional composition of marine communities prior to each mass extinction event. SHAP force plots enable us to explore selectivity patterns for a single functional group at each event and, therefore, serve as a fairer test for comparing extinction selectivity.

**Figure 6.**
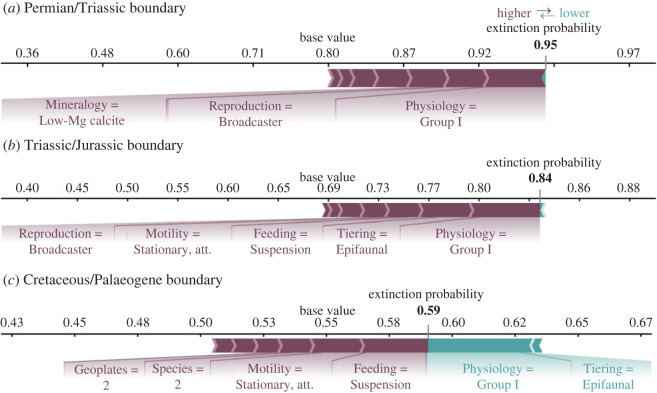
SHAP force plots for each event for Functional Group 1, which comprises articulate brachiopods and bryozoans with the following functional traits: tiering = epifaunal, motility = stationary, attached, feeding = suspension feeder, respiratory protein = hemerythrin, reproduction = broadcaster, mineralogy = low-Mg calcite, skeletal physiology = group I, within genus species = 2, geoplates = 2. (*a*) End-Permian mass extinction, (*b*) end-Triassic mass extinction and (*c*) end-Cretaceous mass extinction.

**Figure 7.**
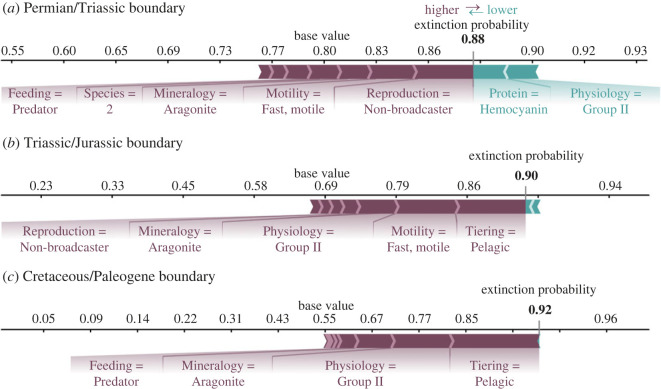
SHAP force plots for each event for Functional Group 2 that comprises cephalopods with the following functional traits: tiering = pelagic, motility = fast, motile, feeding = predator, resporitory protein = haemocyanin, reproduction = non-broadcaster, mineralogy = aragonite, skeletal physiology = group II, within genus species = 2, geoplates = 2. The values for each functional trait are shown in the figure. (*a*) End-Permian mass extinction, (*b*) end-Triassic mass extinction and (*c*) end-Cretaceous mass extinction.

Functional Group 1 (FG1) is one of the most abundant functional groups in our total dataset, and is composed of brachiopods and bryozoans. The SHAP force plot for this group (figure 6) shows that while FG1 was selected against during each event, and each algorithm predicted extinction for this group, the importance of the individual trait categories possessed by this group is different for each event. For example, skeletal physiology is the most important trait for predicting extinction at the end-Permian and end-Triassic events. However, the second-most important trait during the end-Permian is the functional group’s mode of reproduction, while the second-most important trait during the end-Triassic is their tiering. FG2 is the next most abundant in our dataset, which is composed of predatory cephalopod genera. Similar to FG1, FG2 is predicted to go extinct at each event, but the underlying SHAP values for each ecological trait are ranked differently (figure 7). What is consistent, however, is that this group’s aragonitic mineralogy and pelagic tiering are strongly associated with its selective extinction at each event (figure 7). The SHAP force plots confirm the summary SHAP plots in that the selectivity pattern for each extinction event was unique.

## 4. Discussion

### 4.1. Mass extinctions alter selectivity regimes

Our analyses and previous studies (e.g. [38,55]) show that throughout geologic history, geographical range size and within-genus species richness have been the key determinants of extinction at generic level. However, mass extinctions, their aftermaths, and the end-Cenomanian (OAE2) extinctions, served as exceptions to this rule (figure 1). Understanding extinction selectivity during intervals with low extinction rates is difficult because the small number of victims during these time intervals, compared with the much larger numbers of survivors, makes it difficult to find significant relationships or produce a model that can predict the extinctions (see also [34,46,61]). In spite of this, our results show a clear distinction in the traits linked to extinction and survival between these two different extinction regimes.

The importance of geographical range size in extinction selectivity during ‘background’ intervals (those with relatively low extinction rates) is supported by a vast body of previous literature demonstrating that geographical range size is significantly and positively associated with survivorship for most of the Phanerozoic [29,38,55,62–64]. While sometimes dismissed as heavily influenced by sampling bias, a recent simulation study has indicated that the geographical ranges of extinct animals can be reliably reconstructed from the fossil record [65], although using different metrics to the one applied here. Geographical range size is likely to be a good predictor of extinction risk because genera with wide ranges, especially those that span different climate zones, are probably tolerant of a wider range of environmental conditions, and hence are less vulnerable to climate change. Large geographical ranges also buffer taxa from extinction when subjected to stresses affecting limited geographical areas [62]. Mass extinctions and some second-order extinction events, however, do not exhibit geographical range selectivity, a finding supported by previous literature [38,40]. These events are likely to be so severe in their taxonomic losses because of the geographically widespread nature of environmental disturbance during these intervals, meaning that at these times, a large geographical range no longer confers an advantage. This is supported by the extinction of genera with similar ecological and skeletal physiological characteristics, but different spatial distributions across the globe, during such events [38].

Previous research has also shown that during background intervals, rarity (taxa with small geographical ranges, narrow habitat tolerances, small populations or any combination thereof), within-genus species richness and genus age are good predictors of extinction [42,55,62,66]. However, these factors have been shown to positively correlate with one another, and are often calculated in ways which render them interdependent (e.g. number of localities is a proxy for abundance in [66]), making it difficult to determine which is most important in determining extinction risk [42]. The importance of within-genus species richness, as supported by this study, can be explained in that genera with a high number of species would generally be expected to contain a larger pool of genetic and trait variation and, collectively, tolerance of a wider range of environmental conditions [46]. There will, however, be exceptions, as the breadth of environmental tolerances of individual species are highly variable (e.g. [67]).

### 4.2. How predictable are mass extinction events?

Our results show that extinction selectivity is not consistent between the end-Permian, end-Triassic and end-Cretaceous mass extinction events, both in terms of the importance of particular traits (figure 5) and the influence of specific trait states (figure 6). Selectivity models trained on one mass extinction are generally a poor fit to other events (figure 3). Extinction selectivity during mass extinctions, therefore, appears to be unpredictable. It might be expected that functional groups would experience the same outcome (i.e. would preferentially survive or go extinct) during each mass extinction, but this was only true for six of the 31 functional groups present at all three events. In some functional groups, such as FG1, this is because they became increasingly resistant to extinction during each subsequent event (figure 2).

It is reasonable to have expected that the end-Permian and end-Triassic mass extinctions had similar selectivity patterns, as both were caused by LIP volcanism, in contrast to the bolide-impact-driven end-Cretaceous mass extinction. The end-Permian and end-Triassic also occurred only approximately 50 Myr apart, whereas approximately 140 Myr elapsed between the end-Triassic and end-Cretaceous mass extinctions. A likely selective similarity between the end-Permian and end-Triassic mass extinctions has often been asserted in previous literature (e.g. [43,68]), but is rarely thoroughly investigated. We found no evidence of this similarity, with all three mass extinctions showing unique patterns of selectivity. Even though the end-Triassic and end-Cretaceous mass extinctions appear to have been more similar in their extinction selectivity, with tiering and skeletal physiology being the best predictors of extinction (figure 4), the trait states selected for and against were different (figure 5). This result highlights the importance of looking at higher resolution, multi-state characters when investigating extinction selectivity, as previous studies investigating binary trait characteristics of motility, tiering, body size and skeletal physiology have not uncovered this nuance.

One explanation as to why extinction selectivity during mass extinctions is so difficult to predict is that mass extinctions and the diversification events that follow them are highly selective [44,61,69], fundamentally altering the taxonomic and functional composition of the oceans [70–72]. This created stark differences between pre-extinction faunas, shifting the ecological baseline on which each subsequent mass extinction acted (figure 2). A clear shift in the dominant evolutionary fauna occurred across the end-Permian mass extinction [70,71]. The transition from the Mesozoic to Cenozoic faunas occurred throughout the Cretaceous [71,73], a transition attributed to the Mesozoic Marine Revolution (MMR; a predator–prey arms race that stimulated the evolution of animals into functional innovation), which caused an increase in functional space occupation and evenness [54]. The MMR is a key diversification interval because benthic energy budgets are thought to have increased during this time due to changes in primary productivity, and there was a shift towards the ecological dominance of more metabolically active clades, such as predators [74]. The MMR also coincides with the evolution of phytoplankton lineages (nannoplankton, dinoflagellates and diatoms) that fundamentally altered how rapid carbon injection would manifest in the oceans [75]; for example, the evolution of calcareous nannoplankton in the Triassic [76], planktic foraminifera during the Jurassic [77] and diatoms during the Cretaceous [78] would each have hindered the development of widespread ocean acidification. This could explain why we did not see extinction selectivity patterns that might be expected under acidic oceanic conditions, particularly in traits such as mineralogy and skeletal physiology.

Our results indicate a gradual increase in the number of dominant functional groups, and the evenness of their abundances, with each subsequent pre-extinction fauna (figure 2). Functional composition changes occur as genus-rich functional groups experience the highest extinction rates and lose their dominance, while previously subordinate and new functional groups radiate, resulting in the evening out of taxonomic diversity across ecological modes [39,72]. Roopnarine [79] also demonstrated that an increase in ecological complexity (taxonomic and functional richness) leads to an increase in ecosystem resilience to secondary extinctions. Each subsequent extinction event studied here affected a more ecologically complex ecosystem than the last, and this probably influenced the observed contrast in selectivity regimes between them. In addition, Knope *et al.* [72] found that subsequent mass extinctions have resulted in an increased coupling between taxonomic differentiation and modes of life over the course of the Phanerozoic, which in turn has also driven increased functional evenness.

### 4.3. Implications for the present

An important tool for policy making, to try and prevent a projected biodiversity crisis, is being able to make predictions on (i.e. forecast) the extinctions that will occur in the face of future abrupt Earth system changes [3,6,7,80]. If palaeontologists can detect consistent selectivity patterns over geologic time, or link extinctions with certain drivers (e.g. the effects of rapid climate warming) then we can start to make predictions about the relative vulnerability of different clades to extinction in the future. The fossil record presents us with opportunities to examine and compare extinction selectivity during intervals of varying extinction intensity [61,64]. It is clear from our results and previous research [33,38,46,56] that time intervals with low extinction rates and mass extinctions do not record the same selectivity patterns. Traits which may aid survival during most of geologic history may not confer the same advantage during mass extinctions. As a result, a large geographical range and a high number of constituent species may not ensure that a modern genus will be resilient to extinction.

Overall, our results indicate that patterns of extinction selectivity have contrasted between past mass extinctions. Given that models trained on one mass extinction were a poor fit for another, it is not possible for us to use these models to make inferences about future extinctions, at least for marine invertebrates. The baseline functional composition and ecological structure of the oceans on which the mass extinctions were acting was different for each subsequent event, and would also be different for a projected biodiversity crisis. Gaining a better understanding of the state of the modern oceans, particularly developing our knowledge of extant clades that are well represented within the fossil record, may provide insights into which, if any, past mass extinctions acted on a similar functional baseline.

### 4.4. Future research directions

Insight into the predictability of extinction selectivity may yet be gained via studies that focus on individual clades, compare events across a shorter time frame (within which there are no transitions between evolutionary faunas) or consider intervals of intermediate extinction severity. One example is Raja *et al.* [48], which investigated the extinction selectivity of functional traits specific to corals from the Plio-Pleistocene. Their models showed that if selective extinction pressures are consistent with a projected biodiversity crisis, the International Union for Conservation of Nature (IUCN) have probably underestimated the extinction risk of a high proportion of extant corals. Similarly, Tietje & Rödel [47] investigated several traits of living amphibian species with a fossil record and found that species longevity was a good predictor of extinction risk, and that data-deficient species had a high extinction risk. It may be more informative for future research to focus on geological intervals with heightened extinction levels (although not necessarily classed as ‘mass extinctions’) when inferring extinction risk, for example different hyperthermals in the Mesozoic and Cenozoic [15], in order to make the closest comparison with future climate predictions.

An alternative way to potentially improve extinction selectivity models is to add or change which traits are included, but this should also be done with caution. Including too many factors in an analysis can lead to overfitting, and some previous studies have taken the approach of investigating multiple simple models with different variable configurations to avoid this (e.g. [64]). However, both gradient boosting algorithms and recursive feature elimination in the model reduce overfitting of the data. A key functional trait that could be included in future analyses is body size, which is relevant because it controls the rate at which an organism uses energy and resources. In addition, body size has previously been found to be a good predictor of extinction in the marine realm over the Phanerozoic [43,69,81]. Extinction selectivity based on body size has also been found to show different patterns between intervals with low extinction rates and mass extinctions: during intervals with low extinction rates, smaller species are preferentially lost, while during mass extinctions, typically larger species preferentially go extinct [69,82]. Body size changes across mass extinctions are very dynamic (e.g. [83]) and currently there is not enough data on the body size changes of surviving genera to include this factor in a dynamic way in our analysis. As highlighted by Finnegan *et al.* [55], other ecological traits that could potentially be investigated (many of which are important predictors of extinction in modern assessments) include ontogeny, number of births per year, life span, habitat preference and trophic position.

In palaeobiological extinction selectivity studies, the extinction of species is almost always assumed to have been directly linked to significant abiotic stress, such as thermal stress, acidification or anoxia. However, ecological theory suggests that extinction dynamics are most effectively studied within a community framework, where information about biotic interactions among taxa can be taken into account. Such interactions may accentuate or buffer the responses of individual taxa from abiotic (primary) drivers of extinction [84,85]. In fact, many victims of past extinction events are unlikely to have become extinct as a direct effect of abiotic stress, but probably did so in response to cascading ‘secondary’ effects [79,86]. It is likely that some of the extinctions at lower trophic levels observed here, such as selectivity against suspension feeders, can be qualitatively linked to one of the aforementioned abiotic drivers, but other prominent extinction patterns, such as the high levels of extinction amongst predatory taxa, are harder to explain in the absence of extinction cascades. Modelling food web networks could provide insight into biotic influences on extinction dynamics.

## 5. Conclusion

Our functional trait-based model of extinction risk, which used a machine learning algorithm (categorical gradient boosting on decision trees), indicated that extinction selectivity was not consistent in marine invertebrates between the end-Permian, end-Triassic or end-Cretaceous events. This means that predictive models of extinction risk for subsequent mass extinction events could not be generated using our functional trait-based approach. This lack of predictability is attributed to the importance of the taxonomic and functional baseline on which a mass extinction acts, the evolution of new organisms and changes to global biogeochemical cycling and the wider Earth system. In addition, our results show that mass extinctions have unique extinction selectivity patterns to other time intervals, meaning that ‘background’ extinction selectivity signals also cannot be used to make predictive models for mass extinctions. Our analyses indicate that there is no clear set of traits which leave a clade vulnerable during mass extinctions, a result which is of limited utility when inferring extinction risk in the face of projected climate change. However, the fossil record may still provide insight into extinction selectivity within clades and across shorter time scales. Finally, our approach also demonstrates the utility of machine learning algorithms for quantifying extinction selectivity patterns.

## Data Availability

Data and relevant code for this research work are stored in GitHub: https://github.com/PaleoML/mass-extinction and have been archived within the Zenodo repository: https://doi.org/10.5281/zenodo.7646020 [87]. The data are provided in electronic supplementary material [88].
